# A novel and facile green synthesis method to prepare LDH/MOF nanocomposite for removal of Cd(II) and Pb(II)

**DOI:** 10.1038/s41598-021-81095-w

**Published:** 2021-01-15

**Authors:** Roozbeh Soltani, Rasool Pelalak, Mahboubeh Pishnamazi, Azam Marjani, Ahmad B. Albadarin, Shaheen M. Sarkar, Saeed Shirazian

**Affiliations:** 1 Department of Chemistry, Arak Branch, Islamic Azad University, Arak, Iran; 2 Institute of Research and Development, Duy Tan University, Da Nang, 550000 Vietnam; 3 Faculty of Environmental and Chemical Engineering, Duy Tan University, Da Nang, 550000 Vietnam; 4 Faculty of Pharmacy, Duy Tan University, Da Nang, 550000 Vietnam; 5 Department for Management of Science and Technology Development, Ton Duc Thang University, Ho Chi Minh City, Vietnam; 6 Faculty of Applied Sciences, Ton Duc Thang University, Ho Chi Minh City, Vietnam; 7 Department of Chemical Sciences, Bernal Institute, University of Limerick, Limerick, Ireland; 8 Laboratory of Computational Modeling of Drugs, South Ural State University, 76 Lenin prospekt, Chelyabinsk, 454080 Russia

**Keywords:** Environmental sciences, Chemistry, Materials science, Nanoscience and technology

## Abstract

To date, many nanoadsorbents have been developed and used to eliminate heavy metal contamination, however, one of the challenges ahead is the preparation of adsorbents from processes in which toxic organic solvents are used in the least possible amount. Herein, we have developed a new carboxylic acid-functionalized layered double hydroxide/metal–organic framework nanocomposite (LDH/MOF NC) using a simple, effective, and green in situ method. UiO-66-(Zr)-(COOH)_2_ MOF nanocrystals were grown uniformly over the whole surface of COOH-functionalized Ni_50_Co_50_-LDH ultrathin nanosheets in a green water system under a normal solvothermal condition at 100 °C. The synthesized LDH/MOF NC was used as a potential adsorbent for removal of toxic Cd(II) and Pb(II) from water and the influence of important factors on the adsorption process was monitored. Various non-linear isotherm and kinetic models were used to find plausible mechanisms involved in the adsorption, and it was found that the Langmuir and pseudo-first-order models show the best agreement with isotherm and kinetic data, respectively. The calculated maximum adsorption capacities of Cd(II) and Pb(II) by the LDH/MOF NC were found to be 415.3 and 301.4 mg g^−1^, respectively, based on the Langmuir model (pH = 5.0, adsorbent dose = 0.02 g, solution volume = 20 mL, contact time = 120 min, temperature = 25 ℃, shaking speed 200 rpm).

## Introduction

The escalating level of heavy metal released in the aquatic environment is a cause of widespread public health problems associated with environmental pollution, especially water contamination. Heavy metals released through various anthropogenic activities such as leather, cosmetics, electronics, and battery manufacturing industries are one of the primary sources of this worsening environmental pollution^1,2^. In order to decrease the negative impact of heavy metals on the environment, the level of heavy metal pollution in the environment must be minimized. The challenge is to develop appropriate removal techniques for the remediation of heavy metals^3^. Adsorption technique is considered as an energetically efficient, simple, and economical strategy for heavy metals removal^4–9^. For this adsorption-based process to be efficient, it is essential to have porous adsorbents at the nano-/microscale with acceptable adsorption performance^10–14^. Over the past decade, metal–organic frameworks (MOFs), an interesting class of hybrid crystalline nanoporous substances, have quickly developed into one of the most exciting fields of research in material science, physics, chemistry, and interdisciplinary fields^15,16^. The physicochemical properties of MOFs permit their structural features to be finely tuned—based on reticular synthesis—over an extremely broad range. Indeed, several recent works claimed that these materials could be utilized as an ideal platform for the adsorption of pollutants like heavy metals^17–20^.

To date, heavy metal adsorption performance of various kinds of MOFs has been evaluated, primarily on the criteria of (1) a large heavy metal adsorption capacity though this is not necessarily of primary importance to effectively remove heavy metals under practical conditions, and (2) synthesis of a new MOF with a novel porous structure though it is questionable whether the construction of a new adsorbent does not necessarily promise an adsorbent with acceptable performance for practical use. This evaluation strategy led researchers and scientists to design and development numerous targeted MOFs combining highly specific surface area and the existence of specific organic functional groups as adsorption sites that can effectively capture heavy metal anions/cations. On the contrary, a potential limitation to the use of a large number of MOFs for adsorption applications is the sensitivity of them to moisture and aqueous media, as most of them are prone to hydrolysis, which is associated with structural degradation^17,19,20^. Accordingly, another important challenge in using MOFs as heavy metals adsorbents is the synthesis of water-resistant MOFs. Further, little attention has been paid to the recovery and regeneration of such powder-type adsorbents. Soltani et al.^20^ reported that the chemical attachment of MOF nanocrystals (BMZIF20) on ultrathin sheets of Ni-Co layered double hydroxide (Ni_50_Co_50_-LDH) by in situ synthesis protocol could solve the problem of separating fine MOF particles from the aqueous environment. Under this circumstance, the fine MOF particles (with nanometer size) are uniformly attached to the LDH sheets (with micrometer size) and easily separated from the aqueous environment by centrifugation. Although this strategy is a smart option to solve the problem of separation of nanometer-sized MOF crystals from aqueous solutions, the problem of using the toxic methanol solvent in the synthesis process of MOF and its LDH hierarchical nanocomposite (LDH/MOF NC) remains a challenge. Consequently, it is of great importance to choose suitable components for the preparation of LDH/MOF NC as an applicable adsorbent where both LDH and MOF are prepared via a facile, economic, and environmentally friendly method. Ni_50_Co_50_-LDH is a 2D-ultrathin material that could be an excellent option for use as a scaffold for the growth of MOF crystals due to its extended ultrathin sheets, facile production, large-scale synthesis route, and most importantly, use of a non-toxic water-ethylene glycol (EG) solvent mixture instead of conventional toxic organic solvents^20,21^. Moreover, according to the literature^22^, LDH materials themselves possess the ability to adsorb heavy metals, which can help increase the adsorption performance of heavy metals by the LDH/MOF NC.

Recently, Yang et al.^23^ reported a new water-stable MOF labeled as UiO-66(Zr)-(COOH)_2_ which can be prepared through a facile room-temperature synthesis route. It is our view that, compared to other MOFs, this MOF would be the best choice to combine with Ni_50_Co_50_-LDH to produce a hybrid LDH/MOF NC from the environmental point of view, economic justification, synthesis method, and adsorbent structure.

To meet the above criteria, we report for the first time a novel Ni_50_Co_50_-LDH-COOH/UiO-66(Zr)-(COOH)_2_ nanocomposite (LDH/MOF NC) as a promising water-stable adsorbent. Its synthesis especially emphasizes.

**Scheme 1:**
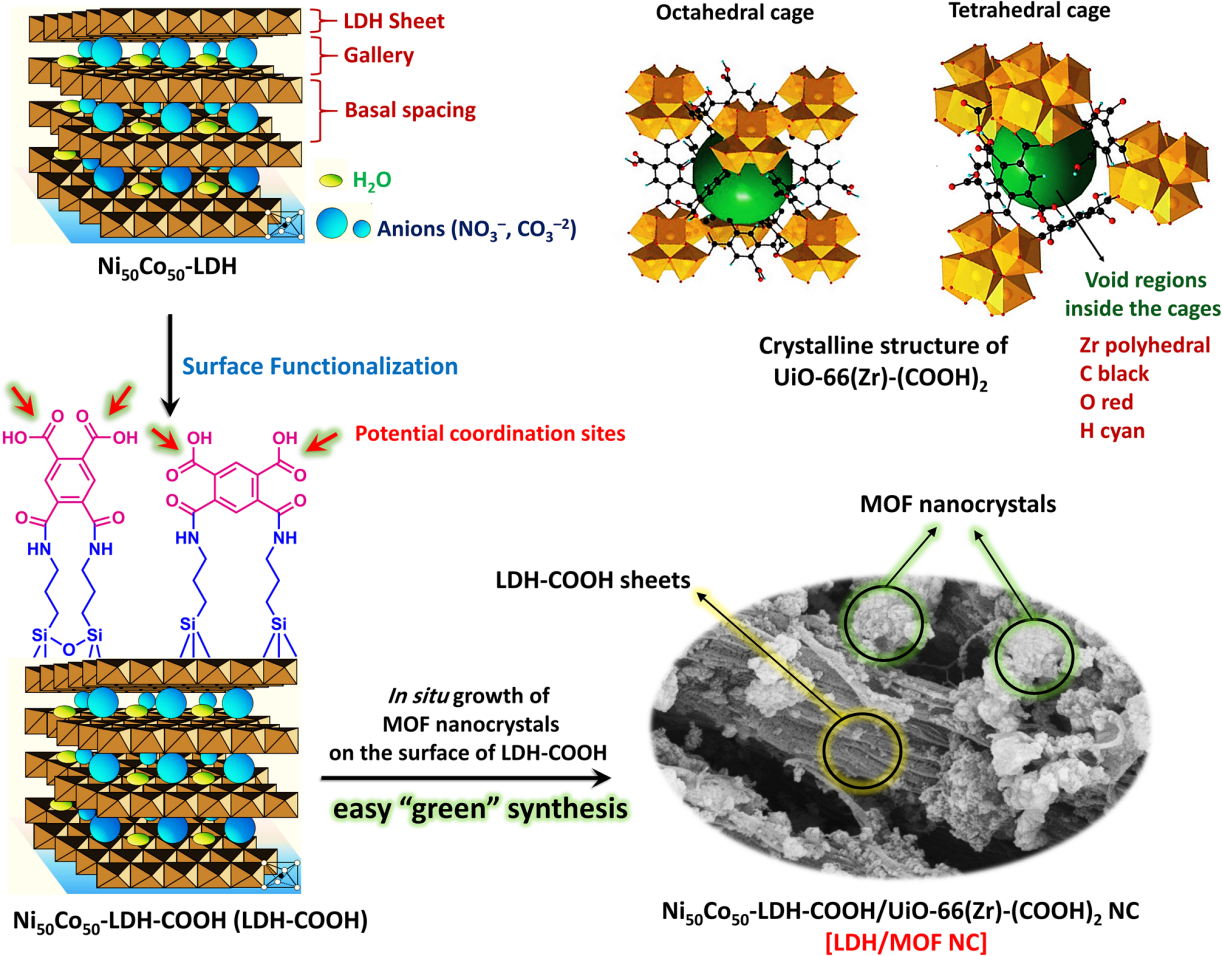
The overall strategy for the synthesis of Ni_50_Co_50_-LDH-COOH/UiO-66(Zr)-(COOH)_2_ NC [LDH/MOF NC].

1. The nature of the solvent: whereas most MOFs and LDHs are synthesized in toxic organic solvents such as DMF, DMSO, methanol, etc., only water was used. In the case of MOF, water has been used both for the preparation and activation process. In the case of LDH, water and EG were used as a solvent mixture. In terms of environmental, economic, and recovery issues, the use of water as a green solvent is a great advantage over organic solvents.
2. The scale: in the case of MOF, a facile ambient-pressure process and a low-cost reaction environment (round-bottom glass flask) was developed, permitting easily scale-up of the production. In the case of LDH, a facile water-EG reflux system was used for large-scale preparation of ultrathin LDH sheets.
3. The introduction of pendent adsorption cites (functional groups) decorating the pores of MOF: free –COOH groups display strong adsorption affinity toward heavy metal cations.
4. Strong covalent bond (chemical attachment) between MOF and LDH surface in the hybrid LDH/MOF NC structure: by implementing a chemical surface functionalization method via attaching –COOH groups on the LDH surface (LDH-COOH), MOF crystals can readily grow on the LDH-COOH surface (Scheme 1). By implementing this synthetic strategy, in addition to being able to use the adsorption properties of both components, we can simplify the separation process (centrifugation) by increasing the particle size of the adsorbent.

## Results and discussion

### Synthesis of LDH/MOF NC

From the point of view of green chemistry, the utilization of safer solvents is important in the preparation of chemical structures. Therefore, in this work, an attempt has been made not to use hazardous organic solvents in the construction of both MOF and LDH, as well as their nanocomposite. Here, for the first time, a synthesis route for the preparation of the LDH/MOF NC has been developed in a green, simple, and functional way, and the synthesized nanocomposite has been used as an adsorbent to remove toxic Cd(II) and Pb(II) heavy metals.

Before in situ growth of MOF crystals on the LDH surface, the surface of LDH nanosheets was functionalized with pyromellitic acid molecules (Scheme 1) to enhance the degree of grafting and enabling coordination bonding between secondary building units in MOF and carboxylate functional groups on the LDH surface (pyromellitic acid molecules). Under this circumstance, UiO-66(Zr)-(COOH)_2_ nanocrystals begin to grow on the surface of Ni_50_Co_50_-LDH-COOH. From the point of view of the formation of a stable nanocomposite structure, surface modification of the LDH nanosheets with carboxylic acid groups is important because it causes a strong chemical interaction between the MOF nanocrystals and the ultrathin nanosheets of LDH and, accordingly, keeps the two components of the nanocomposite together.

### Structure characterization of the samples

In order to study the physicochemical properties of the samples, as well as their structure, the following analyses were used: powder X-ray diffraction (PXRD), Fourier transform infrared (FT-IR), Field emission scanning electron microscopy-energy dispersive X-ray (FESEM-EDX), and N_2_ adsorption–desorption isotherm measurements. Flame atomic absorption spectroscopy (FAAS) analysis was also used to determine the concentration of heavy metals in the aqueous medium.

### XRD characterization

**Figure 1:**
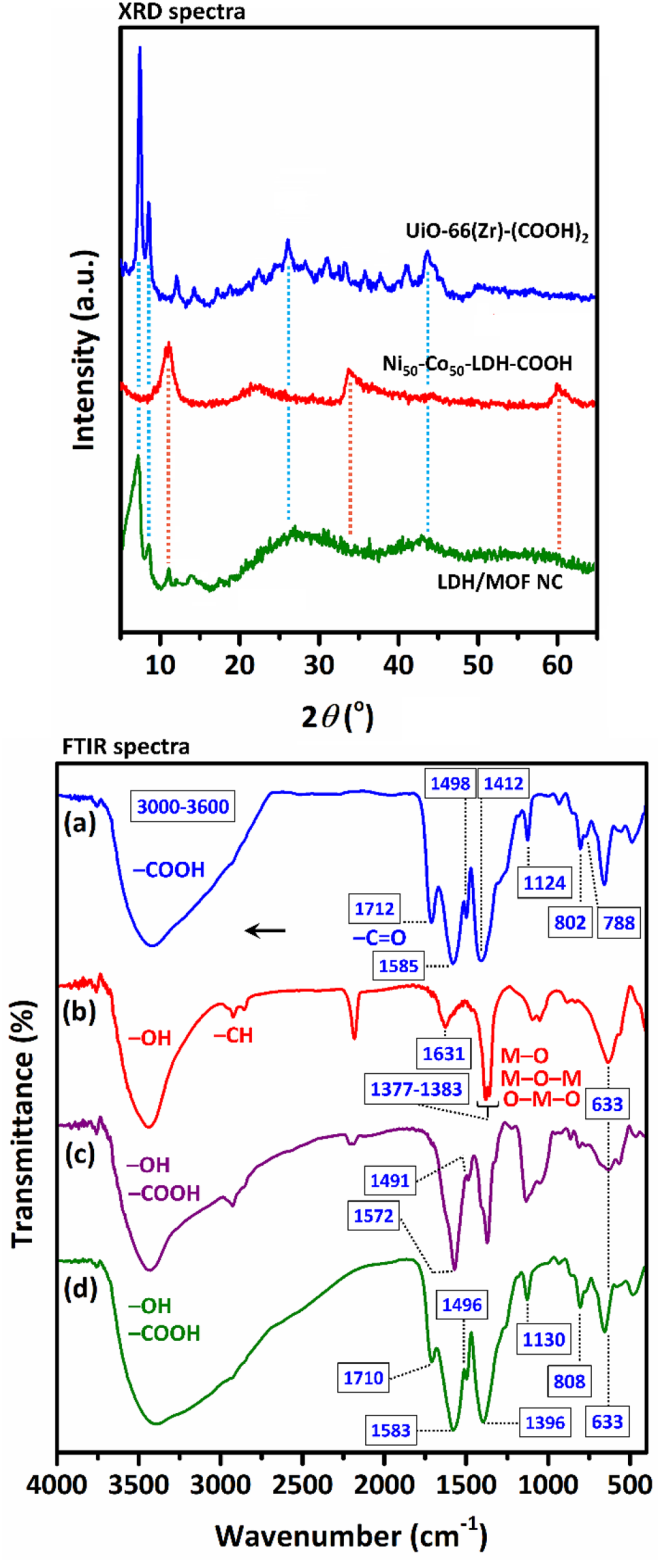
XRD and FT-IR spectra of the samples: (**a**) pure UiO-66(Zr)-(COOH)_2_ (MOF), (**b**) pure Ni_50_Co_50_-LDH, (**c**) Ni_50_Co_50_-LDH-COOH (LDH-COOH), and (**d**) LDH/MOF NC.

As shown in Fig. 1, the XRD profiles of Ni_50_Co_50_-LDH and UiO-66(Zr)-(COOH)_2_ show a similar pattern to the same samples synthesized in previous works which express the successful synthesis of these samples^18,20,21,23–25^. The PXRD pattern of the composite shows that peaks representing both the LDH phase and MOF phase are still present in the composite, indicating the simultaneous presence of both phases in the composite structure and, in other words, the growth of MOF crystals on the LDH sheets. Compared to pure MOF, the crystalline peaks in the composite are less intense due to the simultaneous presence of two phases of LDH (with low crystallinity) and MOF (with high crystallinity) in the composite structure.

### FT-IR measurement

FT-IR spectra of the samples are given in Fig. 1. The characteristic absorption bands related to the functional groups and chemical structure of the pure LDH and MOF were observed and are in agreement with the literature^18,20,21,25^. After surface functionalization of LDH, the presence of new absorption bands at 1572 cm^−1^ and 1491 cm^−1^ was observed which are attributed to the aromatic structure of the pyromellitic acid molecules. Also, a strong broad absorption band in the range of 3000–3600 cm^−1^ region is due to the hydrogen bonding between carboxyl groups. These observations are indicative of successful surface modification of LDH with carboxylic acids groups (LDH-COOH). In the FT-IR spectrum of the composite sample, characteristic absorption bands of both LDH and MOF are observed, indicating the simultaneous presence of both phases in the composite structure. Absorption bands and corresponding functional groups for each sample are listed in Table 1.

**Table 1:** Absorption bands observed in FT-IR spectra of the samples (*OL* overlapped).

Bands (cm^−1^)				
MOF	LDH	LDH-COOH	LDH/MOF NC	Vibrational mode
788		771	788	${\gamma} \text{CH}$ in aromatic rings
802		801	808	Vibrations of phenyl rings
852		860	854	${\delta \text{CO}}({\text{COO}}^{-})$
1124		1133	1130	${\delta \text{CH}}$ in aromatic rings
1412			1396	${{\vartheta }}_{\text{s}}\text{CO }({\text{COO}}^{-})$ in coordinated carboxylic acids
1498		1491	1496	Vibrations of phenyl rings
1585			1583	${{\vartheta }}_{\text{as}}\text{CO }({\text{COO}}^{-})$ in coordinated carboxylic acids
1712		1572	1710	${{\vartheta }}_{\text{as}}\text{CO }({\text{COO}}^{-})$ in free carboxylic acids (pendent)
3000–3600		3000–3600	3000–3600	Crystalline water and acidic OH of carboxylic groups
	633	636	~ 633 (OL)	${\vartheta }$ M–O, M–O–M and O–M–O; M=Co and Ni
	1377–1383	1371–1398	~ 1377–1383 (OL)	Vibration of interlayer ${\text{NO}}_{3}^{-}$ and ${\text{CO}}_{3}^{2-}$ anions
	1631	1630	~ 1631 (OL)	${\delta \text{OH}}$ in water molecules
	2856–2923	2871–2929	~ 2856–2923 (OL)	${{\vartheta }}_{\text{s}}\text{CH}$ vibration (trace amount of EG)
	3446	~ 3445 (OL)	~ 3446 (OL)	${\vartheta }$ M–OH vibration and interlayer water molecules

### FESEM images and EDX mapping analyses

**Figure 2:**
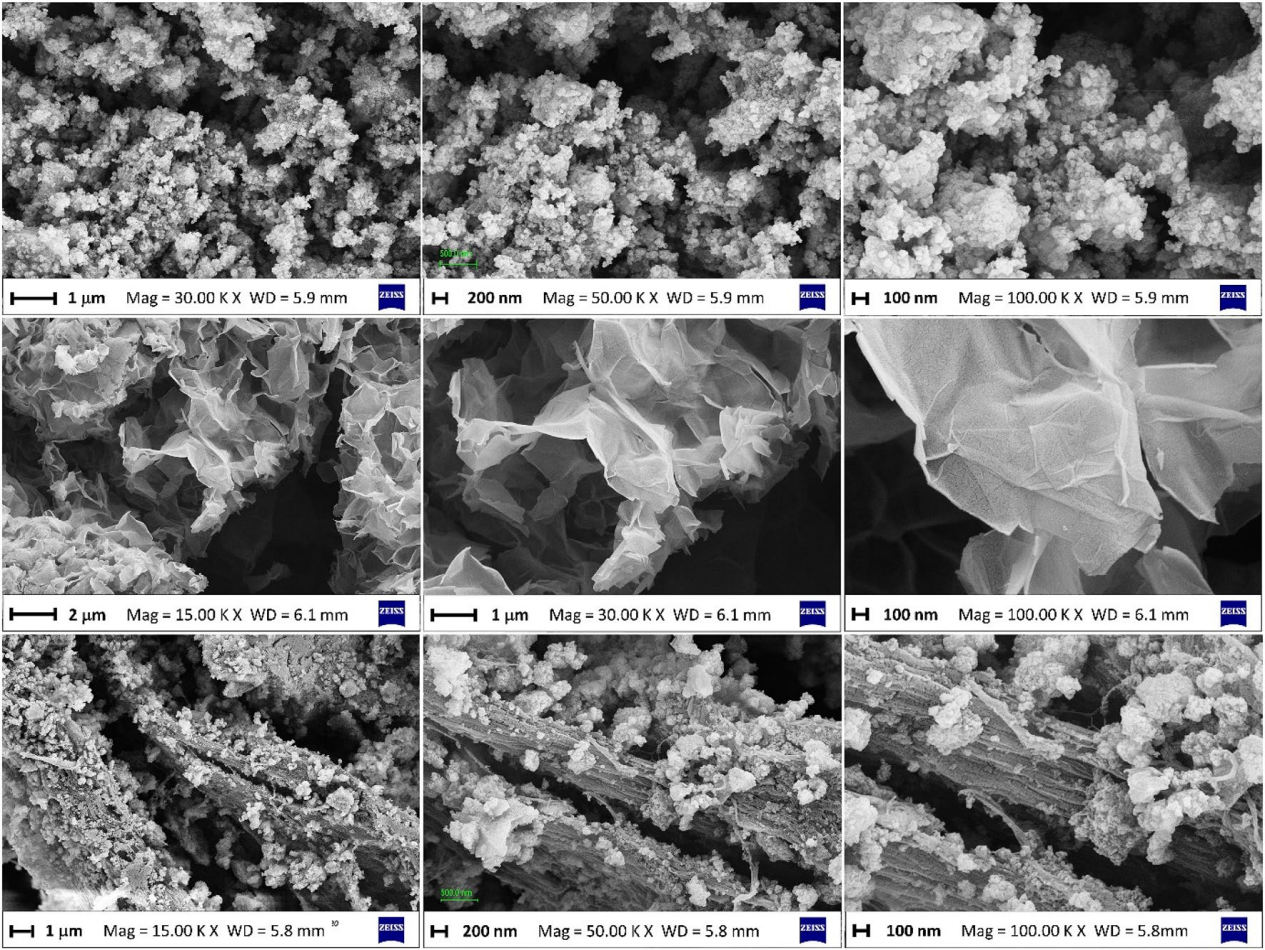
FESEM images of the pure UiO-66(Zr)-(COOH)_2_ (first row), pure Ni_50_Co_50_-LDH (second row), and LDH/MOF NC (third row).

Figure 2 shows the FESEM figures of the samples. Figure 2 (first row) clearly reveals the micrographs of UiO-66(Zr)-(COOH)_2_ nanoparticle. Also, as shown in Fig. 2 (second row), Ni_50_Co_50_-LDH material has an ultrathin sheet-like morphology with a smooth surface. The FESEM images taken after in situ growth of MOF nanocrystals on the surface of LDH sheets clearly show the presence of MOF nanoparticles on the LDH sheets which are uniformly distributed on the sheets (Fig. 2, third row), indicating successful growth of UiO-66(Zr)-(COOH)_2_ nanocrystals on the Ni_50_Co_50_-LDH ultrathin nanosheets. The images plainly demonstrate the role of LDH sheets as a scaffold for the in situ formation and growth of MOF nanoparticles.

**Figure 3:**
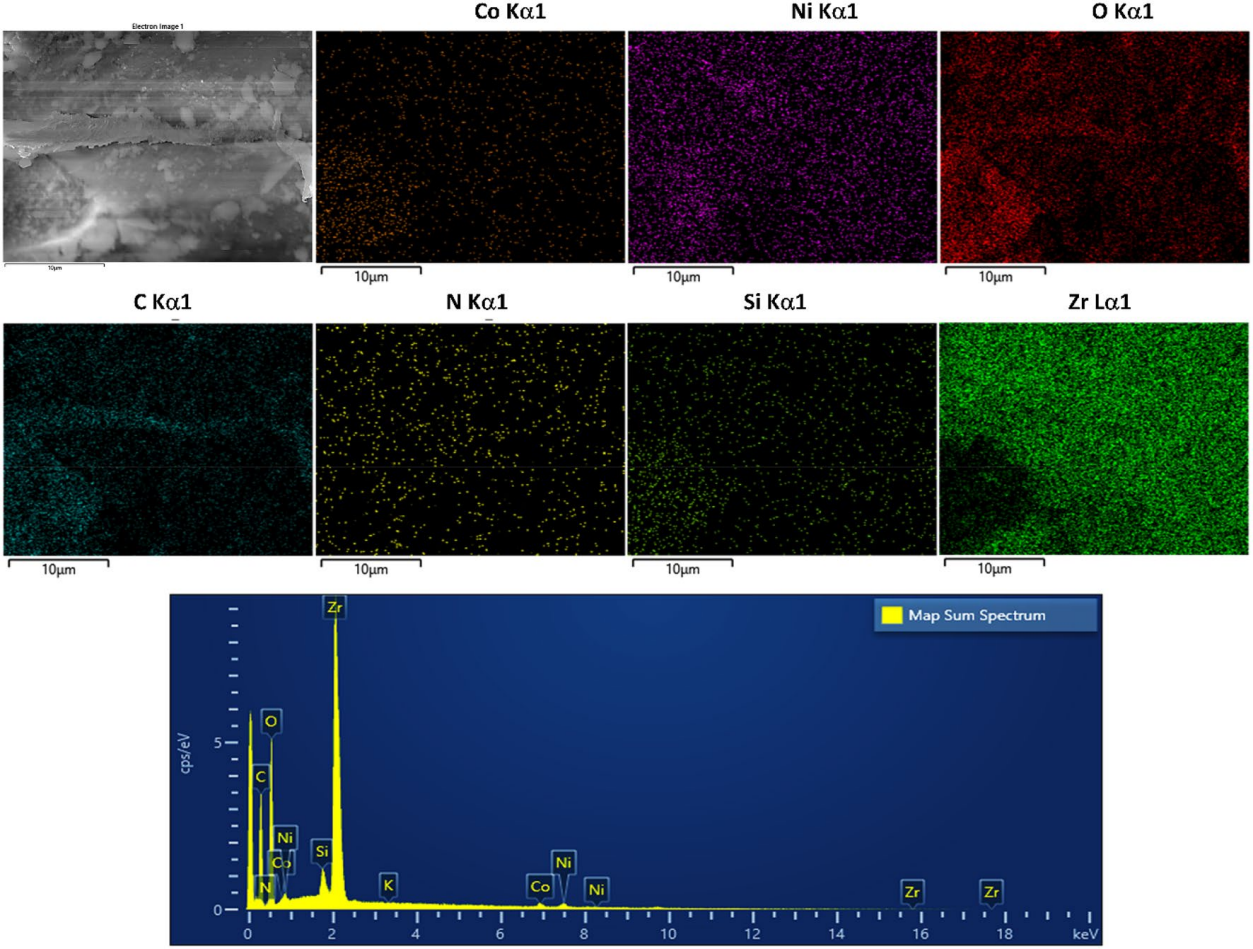
FESEM-EDX mapping images of LDH/MOF NC and the corresponding elements (Co, Ni, O, C, N, Si, and Zr) and its EDX spectrum.

FESEM-EDX mapping of LDH/MOF NC and the corresponding structural elements are shown in Fig. 3. The images reveal that LDH/MOF NC possesses a uniform surface structure with a homogeneous distribution of elements, implying that MOF nanocrystals are uniformly distributed on the LDH ultrathin sheets. Also, the EDX peaks corresponding to the structural elements of composite (Co, Ni, O, N, C, Si, and Zr) are depicted in EDX spectra (Fig. 3). The presence of nitrogen atom in the structure of the composite is due to the presence of (1) silane coupling agent (amine group in APTES molecule) grafted on the LDH surface as well as (2) interlayer nitrate anions (gallery anion) in LDH material (Scheme 1). Also, the presence of Si atom is due to the presence of the silane coupling agent grafted on the surface of LDH sheets (Scheme 1).

### N_2_ adsorption–desorption isotherms

**Figure 4:**
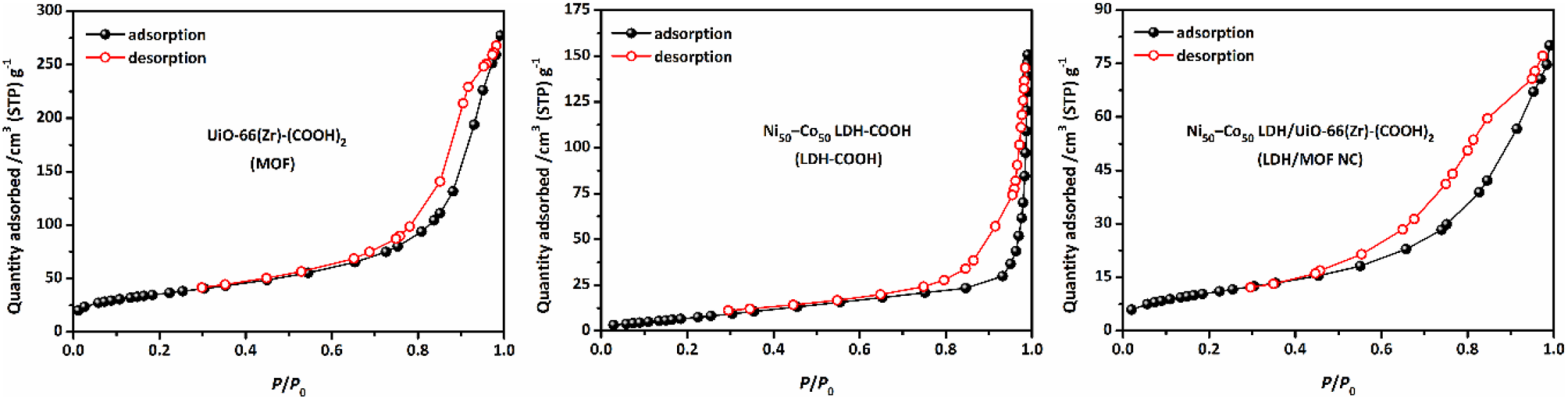
N_2_ adsorption–desorption isotherms of the samples.

**Figure 5:**
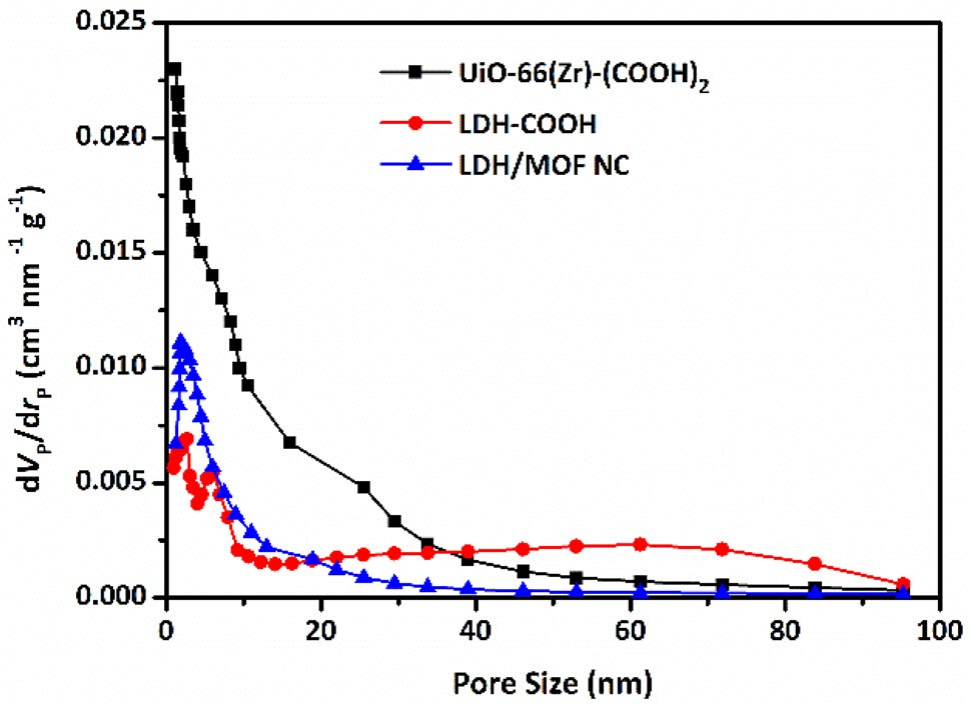
BJH pore size distribution of the samples.

The adsorption–desorption isotherms of the samples were measured with N_2_ gas at 77 K to study their porosity and texture properties. The obtained results are tabulated in Table 2 and corresponding adsorption–desorption isotherms are shown in Fig. 4. The UiO-66(Zr)-(COOH)_2_ exhibits a combination of Type 1(b) and Type IV(a), which is characteristic of microporous and mesoporous material, respectively. Type 1(b) isotherm is the result of a microporous solid having pore size distribution in the micro-meso range, including wide micropores (pore size: 1–2 nm) and possibly narrow mesopores (pore size: < 2.5 nm)^26^. The Ni_50_Co_50_-LDH shows a Type III isotherm with a Type H1 hysteresis loop which is representative of the presence of mesopores and macropores^27^. Adsorption–desorption isotherms of N_2_ for LDH/MOF NC has a similar pattern to UiO-66(Zr)-(COOH)_2_, except that the LDH/MOF NC has a wider hysteresis loop than UiO-66(Zr)-(COOH)_2_, which can be attributed to the presence of larger mesopores in the LDH/MOF NC structure. The presence of such a bi-/trimodal pore system in a porous solid material can facilitate the mass transfer, which in turn can significantly improve the transfer rate of species in the system and, consequently, increase adsorption capacity and reduce equilibrium adsorption time^7,8,17,20,28^. BJH pore size distribution of the samples (Fig. 5) revealed that LDH/MOF NC has a wider pore size distribution than pure UiO-66(Zr)-(COOH)_2_, which is in accordance with the findings from the hysteresis loops.

**Table 2:** The results of BET and BJH analyses for the synthesized samples (${S}_{\text{BET}}$, ${S}_{\text{Langmuir}}$, and ${S}_{\text{Micro}}$ represent surface area according to the BET, Langmuir, and t-plot method, respectively.

Sample	Surface area (m^2^ g^−1^)				PV (cm^3^ g^−1^)	APD (nm)
	${S}_{\text{BET}}$	${S}_{\text{Langmuir}}$	${S}_{\text{Micro}}$	${S}_{\text{Meso}}$		
UiO-66(Zr)-(COOH)_2_	130	122	120	10	0.43	1.2
Ni_50_-Co_50_-LDH-COOH	27	25	15	12	0.23	2.4–6.1
LDH/MOF NC	41	39	32	9	0.13	1.9

${S}_{\text{Meso}}$ and ${S}_{\text{Micro}}$ demonstrate micropore surface area and mesopore surface area, respectively: ${S}_{\text{Meso}}={S}_{\text{BET}}-{S}_{\text{Micro}}$. PV and APD are pore volume and average pore diameters).

### Adsorption studies

Characterization of the LDH/MOF NC structure showed that this hybrid material can be used as an effective adsorbent for the removal of heavy metal species due to its abundant adsorption sites within its porous structure. For this purpose, the adsorption behavior and the removal performance of the synthesized adsorbent for the uptake of Cd(II) and Pb(II) cations were studied. A batch adsorption system for removal of Cd(II) and Pb(II) was used to investigate the adsorption behavior of Cd(II) and Pb(II) on the LDH/MOF NC as an adsorbent. For this purpose, the influence of important adsorption parameters affecting the adsorption performance of the adsorbent was studied at room temperature ($T$ = 25 ℃), including the effect of pH, adsorbent dose, initial metal concentration, and contact time. The heavy metal adsorption capacity on the adsorbent at equilibrium and any time *t*, as well as removal percentage, can be calculated using the following equations^29^:

1 $${Q}_{\text{e}}=({C}_{\text{i}}-{C}_{\text{e}})\times (V/W)$$

2 $${Q}_{\text{t}}=({C}_{\text{i}}-{C}_{\text{t}})\times (V/W)$$

3 $$\%Removal=100\cdot [({C}_{\text{i}}-{C}_{\text{e}})/{C}_{\text{e}}]$$

where ${Q}_{\text{e}}$ and ${Q}_{\text{t}}$ represent the uptake capacity of heavy metals at equilibrium (mg g^−1^) and any time $t$ (mg g^−1^), respectively. ${C}_{\text{i}}$, ${C}_{\text{e}}$, and ${C}_{\text{t}}$ are heavy metal concentration (mg g^−1^) at the initial stage (before adsorption), at equilibrium, and at any time $t$, respectively. $V$ and $W$ parameters are the volume of heavy metal solution (L) and mass of the adsorbent (g), respectively.

**Figure 6:**
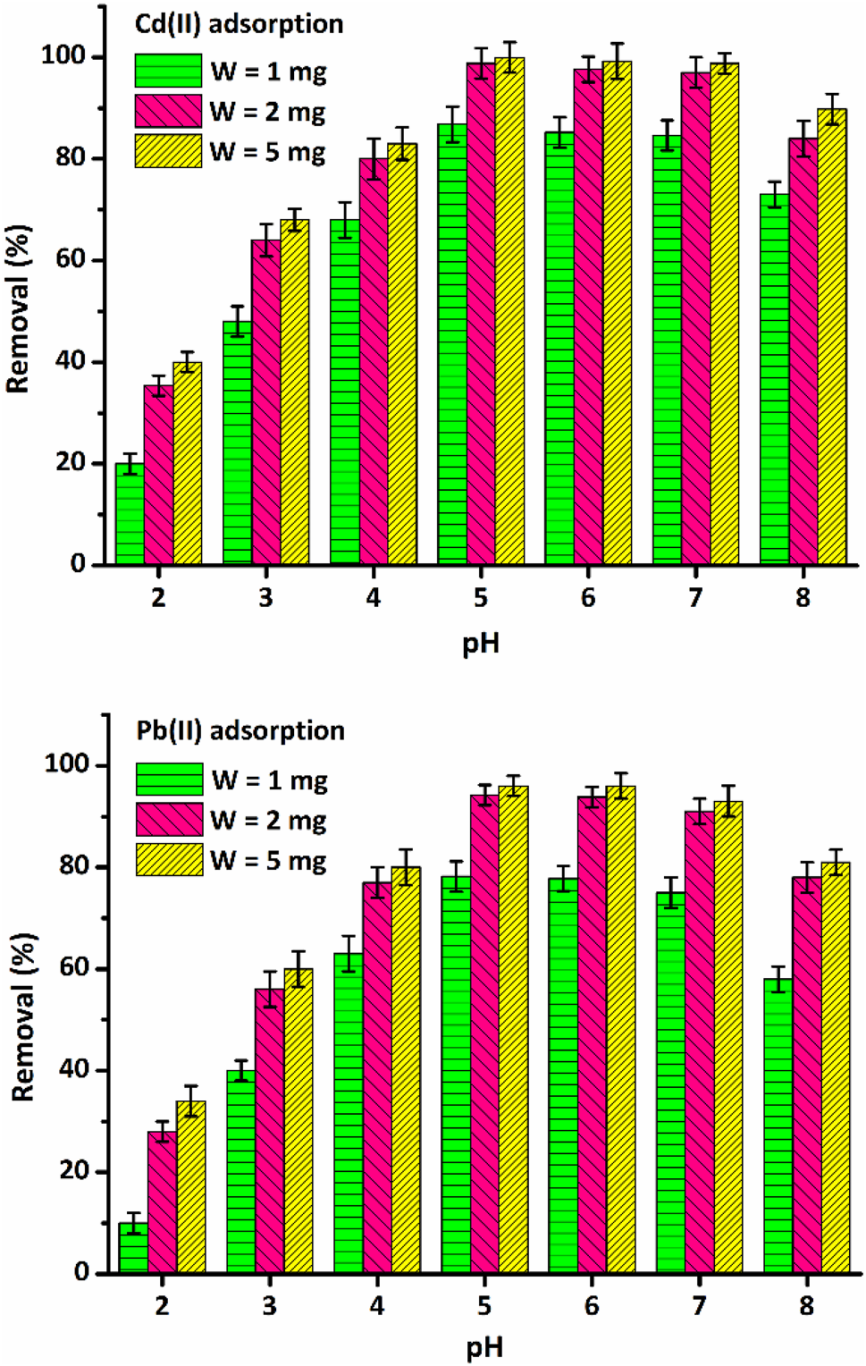
The effect of pH and adsorbent dose on the removal performance of the adsorbent toward Cd(II) and Pb(II) cations ($V$=20 mL, ${C}_{\text{i}}$ = 25 mg L^−1^, $t$ = 120 min, $T$ = 25 ℃, shaking speed 200 rpm).

In the adsorption process, pH and adsorbent dose factors have a direct effect on the amount of adsorption. It has been reported that solution pH has the most significant effect on the physicochemical properties of the adsorbent surface as well as the solution chemistry^30,31^. Therefore, the simultaneous effects of pH and adsorbent dose on the removal of Cd(II) cations by the LDH/MOF NC were monitored and the results are shown in Fig. 6.

For both heavy metals, as the pH increases from 2.0 to 5.0, the removal percentage increases steadily to reach its maximum level (pH 5.0) and then decreases slightly until the pH value of 7.0 above which begins to decrease significantly. This adsorption trend can be seen in all three adsorbent doses. Soltani et al.^12^ have reported that this trend of increasing-maximum-decreasing (IMD) pattern is observed in many cases for the adsorption of cationic heavy metals on adsorbents with active functional groups such as –OH, –SH, –NH, –NH_2_, and –C=O.

In acidic pHs (pH = 2.0–4.0), metal adsorption is low due to the competition between proton (H_3_O^+^) and metal cations for interaction with functional groups (carboxylate groups)^32^. Moreover, according to the previous studies^33^, in low pH environments, low adsorption is observed due to the repulsive interaction between cationic adsorbate species and the positive surface charge of the MOF. Gradually, with increasing pH of the solution and decreasing competition, the adsorption increases to reach its maximum at a certain point, then with further increase in the pH and augmenting the concentration of hydroxide anions, metal cations begin to interact with them, which reduces the adsorption on the adsorbent surface. Zhao et al.^18^ reported that with increasing pH of the solution, the carboxylic form of linkers (–COOH) is converted to carboxylate (–COO^−^), which leads to a stronger interaction between functional groups and heavy metals cations, resulting in increased adsorption. Also, for both Cd(II) and Pb(II), with an increase in the amount of adsorbent from 1.0 to 2.0 mg, the removal percentage increased considerably, but with an increase to 5.0 mg, there was no significant increase in adsorption as shown in Fig. 6. It is reported that because of the stronger electron-accepting affinity of heavy metals like Cd(II) and Pb(II) cations than that of H_3_O^+^ cations, acidic adsorption sites like carboxylic groups can be effective for the capture of metal cations^18^. Here, in acidic pH 5.0 interaction between heavy metals and surface –COOH groups of adsorbent is stronger. Accordingly, pH 5.0 and an adsorbent dose of 2.0 mg were selected as optimum conditions for further adsorption studies.

**Figure 7:**
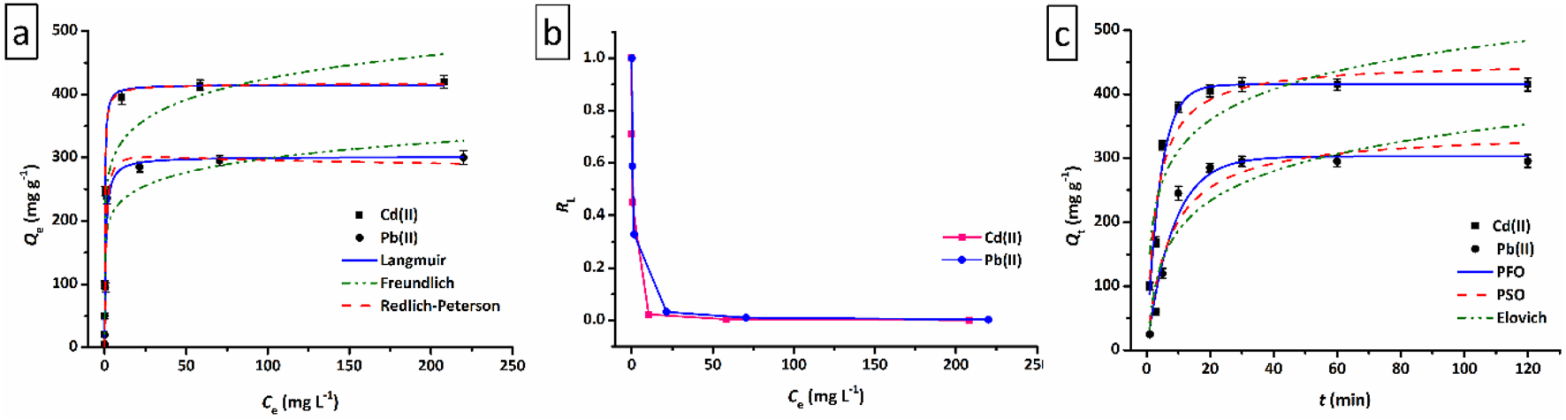
(**a**) The effect of equilibrium metal concentration on the adsorption capacity of Cd(II) and Pb(II) onto the LDH/MOF NC adsorbent and non-linear fitting of experimental data to different isotherm models, and (**b**) corresponding ${R}_{\text{L}}$ values (pH = 5.0, $W$=0.02 g, $V$ = 20 mL, $t$ = 120 min, $T$ =25 °C, shacking speed = 200 rpm); (**c**) The effect of contact time on the adsorption capacity of Cd(II) and Pb(II) onto LDH/MOF NC adsorbent and non-linear fitting of experimental data to different kinetic models (pH = 5.0, $W$ = 0.02 g, $V$ = 20 mL, ${C}_{\text{i}}$ = 100 mg L^−1^, $T$ = 25 °C, shacking speed = 200 rpm).

The effects of heavy metal concentration and contact time on the adsorption process were studied and are depicted in Fig. 7a,c. As shown in Fig. 7a, the changes in the adsorption capacity with equilibrium heavy metal concentration, with increasing heavy metal concentration the adsorption capacity increases dramatically until it is almost fixed at a point (experimental maximum adsorption capacity, ${Q}_{\text{m},\text{exp}.}$ (mg g^−1^)) and the adsorption reaches equilibrium. Also, an almost similar adsorption pattern was observed for adsorption capacity changes with increasing contact time (Fig. 7c).

In order to investigate the adsorption behavior of Cd(II) and Pb(II) cations on the synthesized LDH/MOF NC and to study the possible adsorption mechanism/s in the process different isotherm and kinetic models were fitted to experimental data. The non-linear equations of these isotherms (Langmuir, Freundlich, and Redlich-Peterson (RP)) and kinetics (pseudo-first-order (PFO), pseudo-second-order (PSO), and Elovich) are given as follows^34^:

4 $$\text{Langmuir}: {Q}_{\text{e}}={Q}_{\text{m},\text{cal}.}\cdot [({K}_{\text{L}}\cdot {C}_{\text{e}})/(1+{K}_{\text{L}}\cdot {C}_{e})$$

5 $$\text{Freundlich}: {K}_{\text{F}}\cdot {C}_{\text{e}}^{1/n}$$

6 $$ {\text{R}}{-}{\text{P}}:\left[ {{{\left( {K_{{{\text{R}} - {\text{P}}}} \cdot C_{{\text{e}}} } \right)} \mathord{\left/ {\vphantom {{\left( {K_{{{\text{R}} - {\text{P}}}} \cdot C_{{\text{e}}} } \right)} {\left( {1 + \alpha_{{{\text{R}} - {\text{P}}}} \cdot C_{{\text{e}}}^{g} } \right)}}} \right. \kern-\nulldelimiterspace} {\left( {1 + \alpha_{{{\text{R}} - {\text{P}}}} \cdot C_{{\text{e}}}^{g} } \right)}}} \right] $$

7 $$\text{PFO}: {Q}_{\text{t}}={Q}_{\text{e},\text{cal}.}\cdot [1-\text{exp}(-{k}_{1}\cdot t)]$$

8 $$ {\text{PSO:}}\quad Q_{{\text{t}}} = {{\left[ {k_{2} \cdot Q_{{{\text{e}},{\text{cal}}.}}^{2} \cdot t} \right]} \mathord{\left/ {\vphantom {{\left[ {k_{2} \cdot Q_{{{\text{e}},{\text{cal}}.}}^{2} \cdot t} \right]} {\left[ {1 + Q_{{{\text{e}},{\text{cal}}.}} \cdot k_{2} \cdot t} \right]}}} \right. \kern-\nulldelimiterspace} {\left[ {1 + Q_{{{\text{e}},{\text{cal}}.}} \cdot k_{2} \cdot t} \right]}} $$

9 $$ {\text{Elovich:}}\quad Q_{{\text{t}}} = \left[ {\left( {\frac{1}{\beta }} \right) \cdot (\ln \alpha \cdot \beta ) \cdot t} \right] $$

where, in the Langmuir equation, ${Q}_{\text{m},\text{cal}.}$ and ${K}_{\text{L}}$ represent the calculated maximum adsorption capacity of the adsorbent at equilibrium (mg g^−1^) and Langmuir isotherm constant (L g^−1^), respectively. ${K}_{\text{L}}$ and $n$ are, Freundlich isotherm constant (mg g^−1^) (mg L^−1^)^−1/n^ and a parameter representative of the adsorption intensity (dimensionless) in the Freundlich isotherm, respectively. ${K}_{\text{R}-\text{P}}$ (L g^−1^), ${\alpha }_{\text{R}-\text{P}}$ (mg L^−1^)^−*g*^, and $g$ (dimensionless) are R–P isotherm constants, where $0\le g\le 1$. In the aforementioned kinetic equations, ${Q}_{\text{e},\text{cal}.}$, ${k}_{1}$, ${k}_{2}$, $\alpha$, and $\beta$ are the calculated uptake capacity at equilibrium time (mg g^−1^), the rate constant in the PFO model (min^−1^), the rate constant in the PSO model (g mg^−1^ min^−1^), the initial adsorption rate (mg g^−1^ min^−1^) in the Elovich model, and the adsorption constant (g mg^−1^) in the Elovich model, respectively.

The values of isotherm and kinetic parameters as well as the *R*^2^ values obtained from the non-linear fitting method are given in Table 3. Compared to the Freundlich model, the Langmuir model has a higher *R*^2^ value for adsorption of both Cd(II) and Pb(II) on the adsorbent. Also, the amounts of ${Q}_{\text{m},\text{cal}.}$ (for Cd(II) = 415.3 mg g^−1^ and for Pb(II) = 301.4 mg g^−1^) in the Langmuir model was close to the experimental maximum adsorption capacities (${Q}_{\text{m},\text{exp}.}$ = 420.5 and 300.3 for Cd(II) and Pb(II), respectively), so the Langmuir model is in better agreement with the experimental data and can provide an appropriate isotherm approximation. Based on the Langmuir isotherm, the maximum uptake capacity is related to the complete monolayer coverage on the adsorbent surface^35^. The R–P isotherm can be applied to determine whether the adsorption behavior follows the Freundlich or the Langmuir. In the R–P model, when $g$ = 0 and $g$ = 1 R–P equation becomes the Freundlich equation and the Langmuir equation, respectively^5,8,20^. For adsorption of both Cd(II) and Pb(II), the value of this parameter was very close to unity, demonstrating that the equilibrium data for adsorption of these heavy metals on the LDH/MOF NC fit much better with the Langmuir model than the Freundlich model. The Langmuir model assumes that the adsorption phenomenon takes place on the surface of the LDH/MOF HNC adsorbent with a limited number of identical localized sites via a monolayer coverage.

**Table 3:** The obtained values for isotherm and kinetic parameters using the non-linear fitting method.

Isotherms				Kinetics			
Models	Parameters (unit)	Values		Models	Parameters (unit)	Values	
		Cd(II)	Pb(II)			Cd(II)	Pb(II)
Langmuir	${Q}_{\text{m},\text{exp}}$ (mg g^−1^)	420.5	300.3		${Q}_{\text{e},\text{exp}.}$ (mg g^−1^)	415.0	295.4
	${Q}_{\text{m},\text{cal}.}$ (mg g^−1^)	415.3	301.4	PFO	${Q}_{\text{e},\text{cal}.}$ (mg g^−1^)	415.3	302.6
	${K}_{\text{L}}$(L mg^−1^)	4.073	1.405		${k}_{1}$ (min^−1^)	0.2368	0.1199
	${R}^{2}$	0.9810	0.9515		${R}^{2}$	0.9686	0.9663
Freundlich	${K}_{\text{F}}$ ((mg g^−1^)(L mg^−1^)^1/n^)	245.27	177.54	PSO	${Q}_{\text{e},\text{cal}.}$ (mg g^−1^)	450.4	342.7
	$n$ (–)	8.37	8.85		${k}_{2}$×10^−4^ (g mg^−1^ min^−1^)	7.36	4.17
	${R}^{2}$	0.9074	0.8809		$h$ (g mg^−1^ min^−1^)	149.3	49.0
R–P	${K}_{\text{R}-\text{P}}$ (L g^−1^)	1716.2	371.2		${R}^{2}$	0.9266	0.9147
	${\alpha }_{\text{R}-\text{P}}$ (mg L^−1^)^−g^	4.182	1.087	Elovich	$\alpha$ (mg g^−1^ min^−1^)	610.76	109.94
	g (–)	0.9969	0.9989		$\beta$ (g mg^−1^)	0.0144	0.145
	${R}^{2}$	0.9773	0.9445		${R}^{2}$	0.7640	0.8317

According to Hall et al.^36^ an essential feature of the Langmuir model could be demonstrated in terms of separation factor (${R}_{\text{L}}$) which is a dimensionless equilibrium parameter and suggests the adsorption nature and possibility of the adsorption process: ${R}_{\text{L}}=0$, irreversible;${0<R}_{\text{L}}<1$, favorable;${R}_{\text{L}}=1$, linear; ${R}_{\text{L}}>1$, unfavorable. As shown in Fig. 7b, for the adsorption of Cd(II) and Pb(II) cations on the synthesized LDH/MOF NC adsorbent, the values obtained are between zero and one, implying a favorable adsorption process.

By evaluating the theoretical values of the kinetic parameters obtained after the non-linear fit of the experimental equilibrium data for the adsorption of both Cd(II) and Pb(II), it was found that the PFO and PSO kinetic models have higher values of *R*^2^ than the Elovich models. Also, compared to the PSO model, the PFO model has a ${Q}_{\text{e},\text{cal}.}$ value closer to the ${Q}_{\text{e},\text{exp}.}$ value (${Q}_{\text{e},\text{exp}.}$: experimental adsorption capacity at equilibrium) as shown in Table 3. Consequently, it can be concluded that the adsorption mechanism of Cd(II) and Pb(II) cations on the LDF/MOF NC in an aqueous media is a combination of the PFO and PSO kinetic models with more characteristics of the PFO model, suggesting that the adsorption process takes place initially via the fast response (PFO model). The PFO model points that the rate of adsorption site occupation on the adsorbent is proportional to the number of unoccupied adsorption sites. The PSO kinetic model assumes the rate-limiting step as the formation of a chemisorptive type bond involving sharing or exchange of electrons between surface functional groups of adsorbent and adsorbate.

**Scheme 2:**
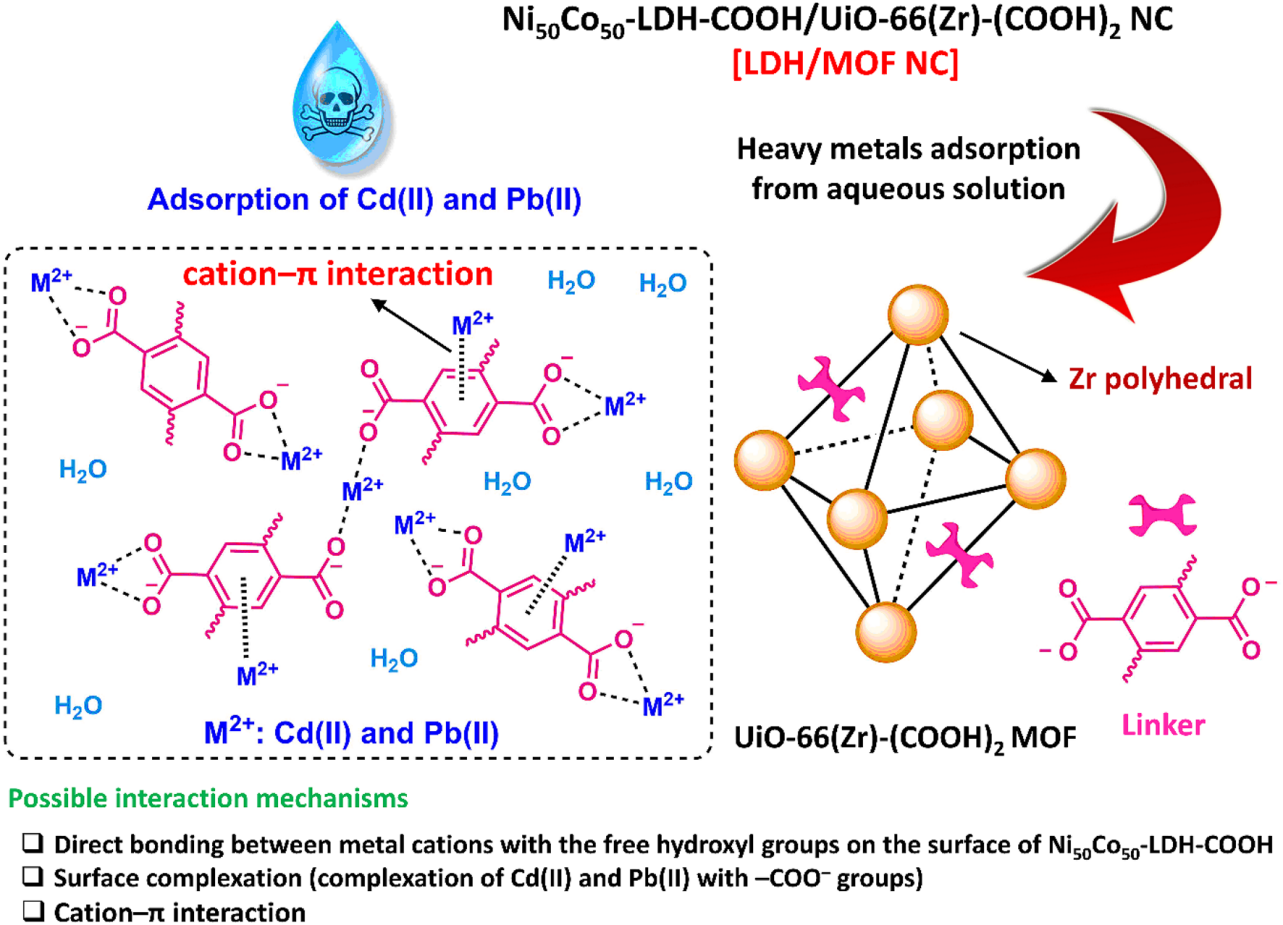
Possible interactions between heavy metals, Cd(II) and Pb(II), and the surface of the Ni_50_Co_50_-LDH-COOH/UiO-66(Zr)-(COOH)_2_ NC adsorbent.

As a result and based on the data obtained from isotherm and kinetic models, it can be suggested that the adsorption mechanism of Cd(II) and Pb(II) on the synthesized LDF/MOF NC is monolayer adsorption on a homogeneous surface with an initial fast adsorption response involving a combination of cation–π interactions (between heavy metal cations and π electron cloud of the aromatic system in MOF structure) and chemisorption involving valency forces through sharing or exchange of electrons (between carboxylate functional groups as complexing functionality and heavy metal cations as well as direct bonding between metal cations with the free hydroxyl and amine groups on the surface of Ni_50_Co_50_-LDH-COOH), as depicted in Scheme 2.

Other plausible mechanisms involve diffusion into micropores and mesopores of the LDF/MOF NC, bulk transport in the liquid phase, mass-transport processes, and diffusion across the liquid film surrounding the LDF/MOF NC.

The most important characteristics of an adsorbent material that directly affect the rate of adsorption and equilibrium adsorption capacity are (1) the degree of porosity and specific surface area, (2) surface functionality and the physicochemical nature of the adsorbent surface, (3) the availability of that surface and its active adsorption sites to adsorbate species, and (4) the morphology and physical size of the adsorbent particles. The Ni_50_Co_50_-LDH-COOH/UiO-66(Zr)-(COOH)_2_ NC synthesized in this study, having all these properties, can play the role of an effective adsorbent for the removal of toxic heavy metal cations from aqueous solution.

### Comparison study

The synthesized LDH/MOF NC possesses a considerably enhanced adsorption capacity for both Cd(II) and Pb(II) cations compared with most LDH-based and MOF-based adsorbents as well as the other nanoporous adsorbents (Table 4). Furthermore, in comparison to other MOF-based and LDH-based adsorbents, as well as the other nanoporous adsorbents, that have been synthesized using toxic organic solvents, the LDH/MOF NC adsorbent was prepared using environmentally friendly solvents such as water and ethylene glycol.

**Table 4:** Adsorption capacities for Cd(II) and Pb(II) by various LDH-based and MOF-based adsorbents as well as the other nanoporous adsorbents (*RT* room temperature, *NR* not reported).

Adsorbents	Res. groups	Year	${Q}_{\text{m},\text{cal}.}$ (mg g^−1^)		Conditions			Ref.
			Cd(II)	Pb(II)	pH	$t$ (min or h^−1^)	$T$ (℃)	
LDH/MOF NC	Soltani et al.	2020	415.3	301.4	5.0	120 min	25	This work
COOH-KCC-1/PA6 NC	Soltani et al.	2020	109.2	–	7.0	240 min	25	^29^
CSt-ZnO	Naushad et al.	2020	–	256.4	6.0	120 min	25	^37^
TATS@AC	Naushad et al.	2020	177.3	–	7.5	240 min	25	^38^
Cu-BTC MOF	Hasankola et al.	2019	–	333	5.0	25 min	RT	^39^
Zn-BTC MOF			–	312	5.0	25 min	RT	^39^
UiO-66-EDTA	Wu et al.	2019	237.2	357.9	5.0	30 min	30	^32^
TA-KCC-1/Chi-OLA NCs	Zarei et al.	2019	–	168	9.0	100 min	25	^4^
TAS-HMSs	Soltani et al.	2019	251.7	295.4	5.0	120 min	25	^12^
M-MCM-41/PVOH NC	Soltani et al.	2018	46.7	–	6.0	240 min	RT	^40^
ZnO nanoflowers	Kataria et al.	2018	71.5	115	6.0	120 min	NR	^41^
CS-LDH	Lyu et al.	2018	140.8	333.3	~ 6.0	60 min	25	^42^
Melamine-MOFs	Yin et al.	2018	–	122.0	5.0	120 min	40	^43^
MOFs			–	72.1	5.0	120 min	40	^43^
UiO-66-NH_2_	Wang et al.	2017	177.3	92.2	6.0	120 min	30	^44^
Cu_3_(BTC)_2_-SO_3_H	Wang et al.	2015	88.7	–	6.0	10 min	RT	^45^
CFR	Naushad et al.	2015	322.6	–	7.0	60 min	25	^46^
DSDH	Shahat et al.	2015	–	169.3	5.2	3 h	RT	^47^
LDH-H	González et al.	2014	~ 39	~ 100	7.0–8.0	48 h	RT	^48^
NH_2_-MCM-41	Heidari et al.	2009	18.3	57.7	2.5–5.0	120 min	25	^49^

*COOH-KCC-1/PA6 NC* carboxylic acid-functionalized fibrous silica KCC-1/polyamide 6 nanocomposite; *CSt-ZnO* starch based ZnO nanocomposite; *TATS@AC* triaminotriethoxysilane grafted oxidized activated carbon; *TA-KCC-1/Chi-OLA NCs* triamine-functionalised mesoporous fibrous silica KCC-1/chitosan-oleic acid nanocomposite; *TAS-HMSs* triamine-functionalized SiO_2_ hollow microspheres; *M-MCM-41/PVOH NC* amino-modified MCM-41/poly(vinyl alcohol) nanocomposite; *CS-LDH* chitosan/Mg–Al-layered double hydroxide nanocomposite; *CFR* curcumin (7-bis(4-hydroxy-3-methoxyphenyl)-1,6-heptadiene-3,5-dione) formaldehyde resin; *DSDH* N,N′di(3-carboxysalicylidene)-3,4diamino-5-hydroxypyrazole.

## Conclusions

Here, for the first time, we have developed a new LDH/MOF NC using a simple, effective, and green in situ approach in a round bottom flask under ambient pressure conditions. MOF nanocrystals (UiO-66-(Zr)-(COOH)_2_) were grafted and grown uniformly over the whole surface of micrometer-sized ultrathin nanosheets of COOH-functionalized LDH (Ni_50_Co_50_-LDH-COOH) in a typical solvothermal condition at 100 °C in a water system. XRD, FT-IR, FESEM-EDX mapping, TEM, and N_2_ adsorption–desorption analyses were applied to characterize and investigate the physicochemical properties of the synthesized samples. The prepared LDH/MOF NC was used as a potential adsorbent for the uptake of Cd(II) and Pb(II) heavy metals cations from aqueous solution and the influence of pH, adsorbent dose, initial metal concentration, and contact time on the adsorption process were investigated. Several non-linear isotherm and kinetic models were applied to find plausible mechanisms involved in the Cd(II) and Pb(II) adsorption, and it was found that the Langmuir and pseudo-first-order models have the best agreement with isotherm and kinetic data, respectively. The calculated maximum adsorption capacities of Cd(II) and Pb(II) by the LDH/MOF NC were found to be 415.3 and 301.4 mg g^−1^ based on the Langmuir isotherm model (pH = 5.0, $W$ = 0.02 g, $V$ = 20 mL, $t$ = 120 min, $T$ = 25 ℃, shaking speed 200 rpm). The results of this study revealed that the LDH/MOF NCs could potentially be used as a promising highly efficient green adsorbent for the removal of toxic metal cations from water.

## Methods

### Materials

All chemicals were used without further purification and are listed in Table 5.

**Table 5:** Chemicals.

Chemical	Purity/grade	Company
1,2,4,5-Benzenetetracarboxylic acid (Pyromellitic acid)	96%	Sigma-Aldrich
Zirconium(IV) chloride (ZrCl_4_)	≥ 99.9%	Sigma-Aldrich
Nickel(II) nitrate hexahydrate [Ni(NO_3_)_2_·6H_2_O]	≥ 98.5%	Sigma-Aldrich
Cobalt(II) nitrate hexahydrate [Co(NO_3_)_2_·6H_2_O]	98%	Sigma-Aldrich
Cadmium nitrate tetrahydrate [Cd(NO_3_)_2_·4H_2_O]	≥ 99.9%	Sigma-Aldrich
(3-Aminopropyl)triethoxysilane	99%	Sigma-Aldrich
Ethylene glycol (EG)	≥ 98.0%	Merck
Ethanol	Absolute and 96%	Merck
Acetone	99%	Merck
Sodium hydroxide pellets (NaOH)	99%	Merck
Hydrochloric acid (HCl)	36%	Merck

### Synthesis of Ni_50_Co_50_-LDH

Following the procedure reported by Soltani et al.^20,21^, in a 1000-mL three-neck round-bottom flask, 5.45 g (18.8 mmol) Ni(NO_3_)_2_·6H_2_O and 10.9 g (37.5 mmol) Co(NO_3_)_2_·6H_2_O were completely dissolved in a mixture of water/EG (112.5 mL/280 mL) under refluxing and vigorous magnetic stirring (90 ℃). After fixing the temperature of the mixture at the desired point, 16.9 g (281.3 mmol) urea was slowly added and the resulting mixture was magnetically stirred under reflux for 3 h (90 ℃). At the end of the reaction time, the mixture was cooled, Buchner-filtered, and repeatedly washed with water and ethanol. Finally, the resulting pale green precipitate was dried at 60 °C for 24 h.

### Synthesis of Ni_50_Co_50_-LDH-COOH (LDH-COOH)

Carboxylic acid-functionalized LDH (LDH-COOH) was synthesized according to the following method: 1.0 g LDH and a certain amount of APTES was introduced into a round bottom flask containing 60 mL ethanol and ultrasonicated for 15 min. Afterward, the mixture was refluxed (24 h), cooled to room temperature, filtered off, washed with ethanol and water, and oven-dried for 24 h. The resulting fine powder was then added to a round bottom flask containing 0.4 g pyromellitic acid and 60 mL ethanol and ultrasonicated for 15 min. The mixture was then refluxed at 160 ℃ for 12 h. Finally, the reaction mixture was cooled, Buchner-filtered, repeatedly washed with water and ethanol, and the resulting precipitate was dried (at 40 °C for 12 h and 100 °C for 12 h).

### Synthesis of UiO-66(Zr)-(COOH)_2_

Pure UiO-66(Zr)-(COOH)_2_ crystals were prepared according to the method developed by Yang et al.^23^.

### Synthesis of Ni_50_Co_50_-LDH-COOH/UiO-66(Zr)-(COOH)_2_ nanocomposite (LDH/MOF NC)

1.0 g LDH-COOH was added into 250 mL round bottom flask containing 100 mL water and ultrasonicated for 15 min. Then, 0.8 g ZrCl_4_ and 1.5 g pyromellitic acid was added to it under stirring and the resulting mixture was refluxed under stirring at 100 °C for 16 h. Finally, the reaction mixture was allowed to cool to room temperature, Buchner-filtered, repeatedly washed with acetone, and the resulting white precipitate was dried at 30 °C for 24 h.

### Batch experiments

An appropriate quantity of analytical grade Cd(II) and Pb(II) metal salt was used to prepare 1000 mg L^−1^ standard stock solution in pure water, and working standards of various concentrations of Cd(II) and Pb(II) were prepared daily by diluting the standard stock solution in pure water.

### Effect of solution pH and adsorbent dose

Various adsorbent doses of LDH/MOF NC ($W$ = 0.01, 0.02, or 0.05 g) were introduced into the stoppered 100-mL polypropylene (PP) bottles containing 20.0 mL aqueous solution with 25 mg L^−1^ of Cd(II) and Pb(II) cations. The pH of the solution ranged from 2.0 to 8.0 and a 0.1 mol L^−1^ NaOH or 1.0 mol L^−1^ HCl was utilized to adjust the pH of the solution throughout the adsorption tests when desired. The PP bottles were then transformed into an incubator shaker with a thermostat and vibrated at 200 rpm and 25 ℃ for 120 min. Finally, the samples were centrifuged at 4500 rpm for 5 min to isolate the LDH/MOF NC particles and the supernatant solution analyzed using FAAS to determine the residual Cd(II) and Pb(II) concentrations.

### Adsorption isotherms and kinetics

For the isotherm tests, the initial volume of the Cd(II) and Pb(II) solutions was 20 mL containing different concentrations of Cd(II) and Pb(II) cations (0.5–250 mg L^−1^) and 0.002 g of LDH/MOF NC particles. The PP bottles were then transformed into an incubator shaker with a thermostat and vibrated at 200 rpm and 25 ℃ for 120 min. Finally, the samples were centrifuged at 4500 rpm for 5 min to isolate the LDH/MOF NC particles and the supernatant solution analyzed using FASS to determine the residual Cd(II) and Pb(II) concentrations. For kinetics testing, the conditions are similar to the above trend except that the initial concentration of heavy metals is 100 mg L^−1^, and the contact time varied between 1 and 120 min.

### Characterization

The instruments used to characterize the structures of the samples as well as to determine adsorbate concentrations are listed in Table 6.

**Table 6:** Instruments and methods.

Characterization method/instrument	Model/company	Country
Fourier transform infrared (FT-IR)	Avatar 370, Thermo Nicole	USA
Field emission scanning electron microscopy-energy dispersive X-ray (FESEM-EDX)	SIGMA HV, Zeiss	Germany
N_2_ adsorption–desorption isotherm measurements (porosimetry analyzer)	Belsorp-mini II, BEL	Japan
Powder X-ray diffraction (PXRD)	AW-XDM300, Asenware	China
Flame atomic absorption spectroscopy (FAAS)	PerkinElmer Model A300	Norwalk, USA
